# How to correctly estimate the electric field in capacitively coupled systems for tissue engineering: a comparative study

**DOI:** 10.1038/s41598-022-14834-2

**Published:** 2022-06-30

**Authors:** João Meneses, Sofia Fernandes, Nuno Alves, Paula Pascoal-Faria, Pedro Cavaleiro Miranda

**Affiliations:** 1 Centre for Rapid and Sustainable Product Development from the Polytechnic of Leiria, Leiria, Portugal; 2 Mathematics Department of Faculty of Management and Technology from the Polytechnic of Leiria, Leiria, Portugal; 3 IBEB, Faculdade de Ciências, Universidade de Lisboa, 1749-016 Lisboa, Portugal

**Keywords:** Computational biophysics, Microbiology techniques, Biophysical methods

## Abstract

Capacitively Coupled (CCoupled) electric fields are used to stimulate cell cultures in Tissue Engineering. Knowing the electric field (E-Field) magnitude in the culture medium is fundamental to establish a relationship between stimulus strength and cellular effects. We analysed eight CCoupled studies and sought to corroborate the reported estimates of the E-Field in the culture medium. First, we reviewed the basic physics underlying CCoupled stimulation and delineated three approaches to estimate the E-field. Using these approaches, we found that the reported values were overestimated in five studies, four of which were based on incorrect assumptions. In all studies, insufficient information was provided to reproduce the setup exactly. Creating electrical models of the experimental setup should improve the accuracy of the E-field estimates and enhance reproducibility. For this purpose, we developed a free open-source tool, the E-field Calculator for CCoupled systems, which is available for download from an internet hosting platform.

## Introduction

In tissue engineering (TE), the application of electric fields (E-Fields) to cell cultures has been shown to promote a variety of cellular responses, such as proliferation, migration, growth, differentiation, extracellular matrix expression, or even apoptosis. E-fields are thought to transiently change membrane polarization and permeability of certain ion channels, modulating cascates of events at intracellular level^1^. However, the mechanisms of action of electrical stimulation are still not completely understood^2,3^. This is due, in part, to the fact that it is difficult to compare and piece together the results from different studies because they use different experimental protocols and setups. Accurate estimates of the E-field are essential to compare results from different studies and establish a relation between stimulus characteristics and specific cellular effects. However, this quantity is rarely measured and often estimated with incorrect assumptions of the underlying physics.

In cell stimulation, electrical energy can be transferred to the culture medium by conductive, capacitive or inductive coupling^2^. In the first case, two parallel metal electrodes are placed in direct contact with the culture medium, hence the term direct coupled (DCoupled) to describe this procedure. The main disadvantage of this simple setup is that unwanted chemical species may be produced at the electrode-electrolyte interface, particularly when a constant voltage (or current) is applied. This problem can be minimized by using salt-bridges to connect the electrodes to the culture medium^4^. In capacitive coupling, two typically parallel flat metal electrodes are separated from the culture medium by an insulating layer and no electron transfer reactions occur at the insulator-electrolyte interface^2^. One drawback of capacitively-coupled (CCoupled) systems is that the voltage drop across the culture medium is only a small fraction of the applied voltage, i.e., the E-Field induced in the culture medium is weak compared to that obtained by direct coupling. However, the efficiency of capacitive coupling increases with frequency and so the strength of the induced E-Field can be increased by working at higher frequencies, as an alternative to applying higher voltages. In inductive coupling, the (insulating) container with the culture medium is placed close to a coil, or coils, carrying a current. A time-varying current flowing in the coil creates a time-varying magnetic field, which in turn induces a time-varying E-Field, a phenomenon known as electromagnetic induction^2^. The amplitude of the E-Field induced in the culture medium by inductively-coupled (ICoupled) systems increases with frequency. The advantages and disadvantages of this method are similar to those already mentioned for capacitive coupling. One aditional reported disadvantage is that the observed cellular effects result from a superposition of electric and magnetic fields. In that case the transformer like coupling (TLC) method can be used to isolate cellular effects due only to an E-Field^5^.

We focused our analysis on CCoupled systems because they avoid the faradaic products that constitute a major disadvantage of DCoupled systems, and the concomitant application of a magnetic field that characterizes ICoupled systems. An extremely large span of E-Field strengths in the culture medium has been reported for CCoupled setups alone, ranging from 1.0 $\times$ $10^{-5}$ V $\hbox {m}^{-1}$^6^ to 1.7 $\times$ $10^{5}$ V $\hbox {m}^{-1}$^7^. In these studies, different methods were used to estimate the E-Field strength, some of them clearly flawed. Yet, they all describe a positive effect of electrical stimulation on cell culture, and some of them are widely cited. The aim of this study is to determine the validity of the E-Field strength estimated in several CCoupled in vitro studies by comparing it with the predictions from three different modelling approaches. We aim also to present a solid methodology to estimate the E-field in CCoupled stimulation protocols, thus contributing to enhance reproducibility and helping to establish guidelines when CCoupled systems are used in TE applications.

## Methods

### Electric circuit model of CCoupled experimental setups

**Figure 1:**
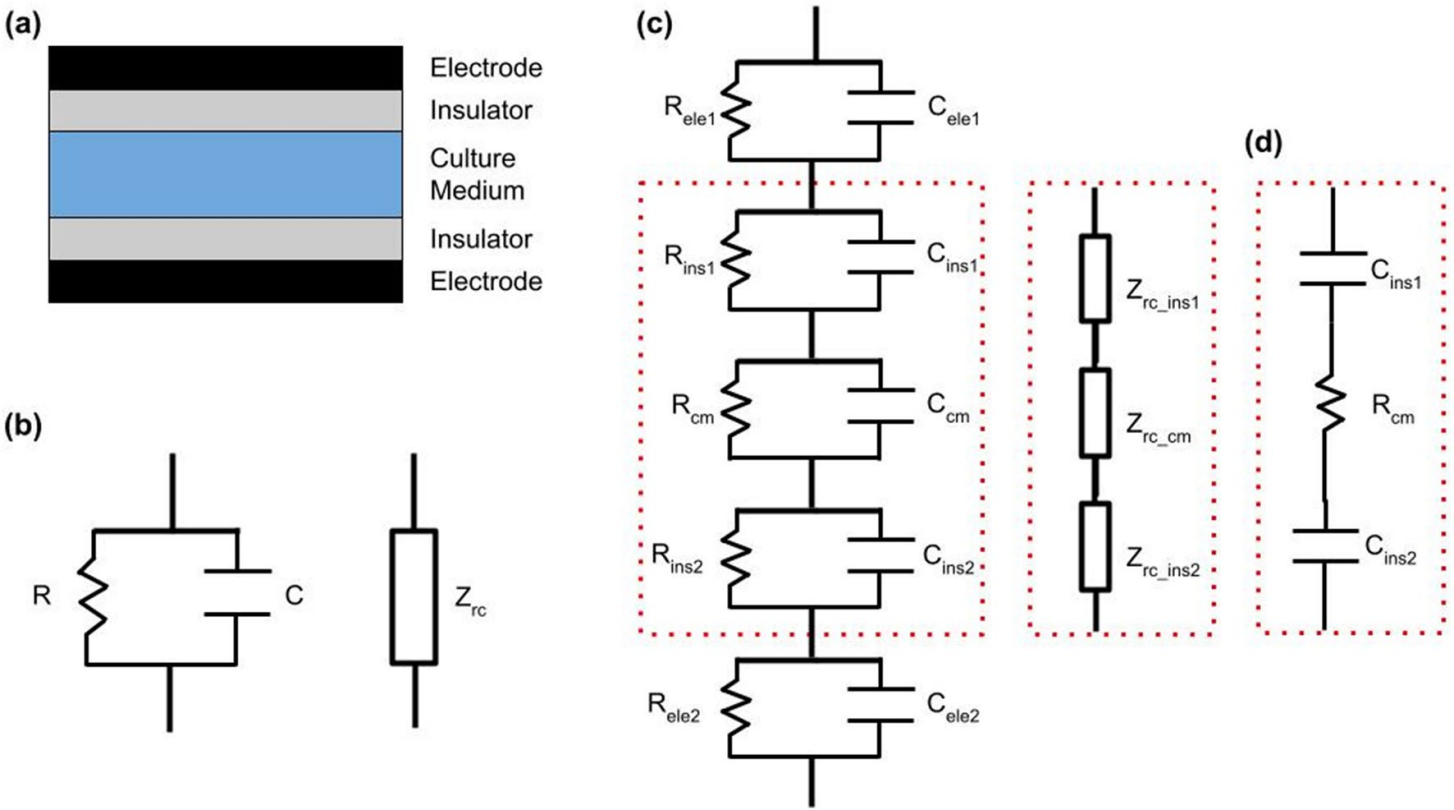
(**a**) Layered cylindrical model of a typical CCoupled experimental setup; (**b**) Resistor-capacitor (RC) model and impedance circuit model for an individual layer; (**c**) RC and impedance circuit models for the five layers; (**d**) The complete circuit can be reduced to its three most relevant components without significant loss of accuracy. Abbreviations: ele-electrode, ins-insulator, cm-culture medium, r-resistor, c-capacitor, z-impedance.

The geometry of experimental setups for capacitively-coupled electrical stimulation of cells can often be modelled as a cylindrical layered geometry, with two circular metallic plates separated from the culture medium by electrically insulating layers, typically plastic, glass or air, as shown schematically in Fig. 1a. Note that all layers have the same diameter in this model. Given the geometry of the setup, electric charge flows parallel to the axis of the cylinder. In general, each layer can be described in terms of a resistor and a capacitor in parallel (Fig. 1b) since both paths are available for flow of charge.

In this geometry, the E-Field within each layer is uniform so the resistance, $R_i$, and capacitance, $C_i$, of the $i{th}$ layer are given by the familiar formulae,

1 $$\begin{aligned} R_i = \rho _i \frac{l_i}{A} \end{aligned}$$

2 $$\begin{aligned} C_i = \frac{\epsilon _{0} \epsilon _{r_i} A}{l_i} \end{aligned}$$

where $\rho _i$ is the resistivity of the material, $\epsilon _{r_i}$ its relative permittivity and $\epsilon _{0}$ the permittivity of vacuum, *A* is the cross-sectional area of the cylinder and $l_i$ the thickness of the layer. The relation between the current, *I*, which is the same in all layers due to charge conservation, and the voltage drop in each layer, *V*, is given by Ohm’s law

3 $$\begin{aligned} V_i = Z_i I \end{aligned}$$

where $Z_i$ is the impedance of the $i{th}$ layer. Note that this is the impedance of the resistor, $Z_r$, and of the capacitor, $Z_c$, in parallel, i.e.,

4 $$\begin{aligned} \frac{1}{Z_{i}} = \frac{1}{Z_{r_i}} + \frac{1}{Z_{c_i}} \end{aligned}$$

The impedances of the resistor and capacitor are:

5 $$\begin{aligned} Z_{r_i} = R_i \end{aligned}$$

6 $$\begin{aligned} Z_{c_i} = j X_{c_i} = -\frac{j}{\omega C_i} \end{aligned}$$

where $X_{c_i}$ is the capacitive reactance, $\omega$ is the angular frequency of the applied sinusoidal signal, and *j* is the imaginary unit. Thus

7 $$\begin{aligned} \frac{1}{Z_{i}} = \frac{1}{R_i} + j \omega C_i \end{aligned}$$

or

8 $$\begin{aligned} Z_{i} = \frac{R_i (1 - j \omega C_i R_i)}{1 + \omega ^{2} C_i^{2} R_i^{2}} \end{aligned}$$

Note that impedances are complex numbers, and that the impedance of a capacitor is frequency dependent, it decreases with increasing frequency.

The whole setup can be viewed as a series of five parallel RC circuits, one for each layer (Fig. 1c)^8^. The total impedance of the five layers in series is the sum of the individual (complex) impedances,

9 $$\begin{aligned} Z_{total} = \sum _{i} Z_i. \end{aligned}$$

The ratio between the applied voltage, *V*, and the current, *I*, through the setup is given by the total impedance, $Z_{total}$, of the setup, i.e.

10 $$\begin{aligned} V = Z_{total} I. \end{aligned}$$

The voltage drop across a single layer, $V_i$, can therefore be obtained as a fraction of the applied voltage

11 $$\begin{aligned} V_i = Z_{i} I = \frac{Z_i}{Z_{total}} V. \end{aligned}$$

Then, the magnitude of the E-Field in a layer is given by

12 $$\begin{aligned} |E_i |= \frac{|V_i |}{l_i} \end{aligned}$$

where $l_i$ is the thickness of the *i*th layer.

The purpose of this section was to show that for simple geometries like the one considered here it is possible to predict the E-Field in the culture medium exactly provided that the physical parameters of the setup are known, namely the dimensions $A_i, l_i$ and electrical properties $\rho _i, \epsilon _{r_i}$, and that the applied voltage has a sinusoidal waveform, which is characterized by a single frequency. In fact, the E-Field in the various layers is independent of the (constant) cross-sectional area *A* because the total impedance of every layer is inversely proportional to *A* and the E-Field is proportional to a ratio of impedances.

**Figure 2:**
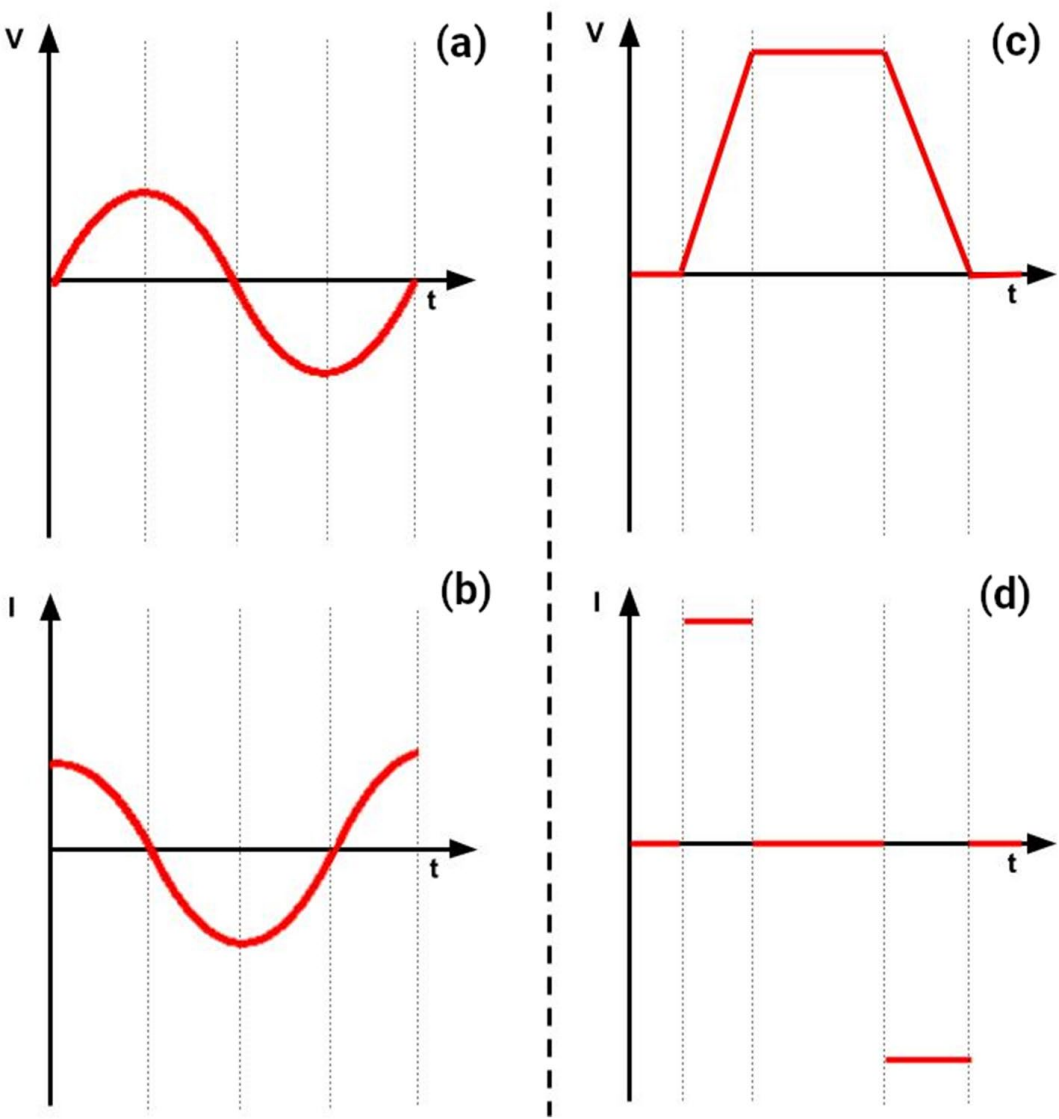
Voltage/current waveforms for a purely capacitive circuit. For an input sinewave voltage (**a**), the resulting current waveform is given by (**b**). For an input trapezoidal voltage (**c**), the resulting current waveform in the circuit is (**d**).

The model shown in Fig. 1c is also useful to understand some general features of CCoupled setups. It turns out that, in any one layer, the impedance of either the resistive or the capacitive arm is much larger than that of the other arm. For the insulating layers $Z_{c_i} \ll Z_{r_i}$, whereas for the conductive layers $Z_{r_i} \ll Z_{c_i}$ (see Table 2 in Results section). In these cases, the equation for the impedance of the *i*th layer (Eq. 4) becomes $Z_i = Z_c$ or $Z_i = Z_r$, respectively. In other words, in insulating layers charge flows almost exclusively through the capacitor whereas in conductive layers charge flows almost exclusively through the resistor. In addition, the impedance of the electrodes is very low due to the high conductivity of metals, so the voltage drop across them can be neglected. As a result of these considerations, the circuit in Fig. 1c can be represented, to a very good approximation, by a capacitor, a resistance, and a second capacitor in series, as in Fig. 1d and in the work of Fitzsimmons et al., Fig. 2^6^ . The capacitors represent the insulating layers and the resistor the culture medium.

In setups commonly used in tissue engineering the impedance of the culture medium, $Z_r$, is much lower than that of insulating layers, $Z_c ,i.e., Z_r \ll Z_c \simeq Z_{total}$. Consequently, the voltage drop across the culture medium is only a very small fraction of the applied voltage (see Eq. 11) and the E-Field in the culture medium is weak. This E-Field can be increased by reducing the impedance of the insulating layers, and hence the total impedance, either by decreasing the thickness of the insulating layers or by working at higher frequencies. It also follows from the equations presented above that, for a fixed capacitive impedance, the E-Field in the culture medium is practically independent of its height (reported as “thickness” in the tables ahead), provided that $Z_r \ll Z_c$. This is because the impedance of the culture medium is proportional to the thickness of the layer, to a very good approximation (Eq. 1), and the E-Field is proportional to the impedance (Eq. 11) and inversely proportional to the thickness of the layer (Eq. 12).

Another important consequence of the low relative impedance of culture medium ($Z_r \ll Z_c \simeq Z_{total}$) is that the total impedance of the circuit is approximately equal to the impedance of the insulating layers. As a result, the circuit responds almost as a capacitor. For a capacitor with capacitance *C*, the relation between current and voltage is given by

13 $$\begin{aligned} I = C \frac{\partial V}{\partial t}, \end{aligned}$$

which is obtained by differentiating $Q = CV$ with respect to time, where *Q* is the charge stored on the capacitor. The E-Field anywhere in the setup is proportional to the current *I*, so its magnitude is determined primarily by the rate of change of the applied voltage. For a sinusoidal applied voltage, the E-Field in the culture medium will also be sinusoidal with a phase lead of approximately 90° and a magnitude that is proportional to the product of the frequency of the sine wave and of its amplitude (Fig. 2a,b). In the case of a trapezoidal pulse, the E-Field will be non-zero only during the risetime and falltime of the pulse and is zero during the plateau. For a linear ramp, the E-Field will be proportional to the amplitude of the wave divided by the rise or fall time. Note that the rising and falling edges of the trapezoidal pulse will produce E-Fields with opposite directions (Fig. 2c,d).

More detailed information about the theory of AC circuits may be found in Physics or Electrical Engineering textbooks, [e.g.,^9^Subchapters 7.2–7.4].

### Numerical approaches for calculating the electric field

#### Analytical

The electrical circuit model described in the previous section can be used to calculate the E-Field in the culture medium for a cylindrical geometry and a sinusoidal applied voltage. The described equations can be easily implemented in Excel, Matlab or Python, for example. When the waveform is not sinusoidal, an estimate of the maximum E-Field strength can be obtained by considering a frequency such that the maximum rates of change with time of the actual voltage waveform and of a sinusoidal waveform of equal amplitude match. For example, for a linear ramp of amplitude *A* and risetime $\tau$ consider a sinusoidal voltage of amplitude *A* and frequency *f*. Then, equating the maximum rates of change of these two waveforms gives the matching frequency:

14 $$\begin{aligned} \frac{A}{\tau } = 2 \pi f A \quad or \quad f = \frac{1}{2 \pi \tau } \end{aligned}$$

The proposed analytical approach will yield estimates of the E-Field of the right order of magnitude even when the geometry is non-cylindrical, but care must be taken to choose equivalent dimensions for the cylindrical model appropriately. Specifically, the thickness of the insulating layers in the cylindrical model should be the same as in the original setup.

**Figure 3:**
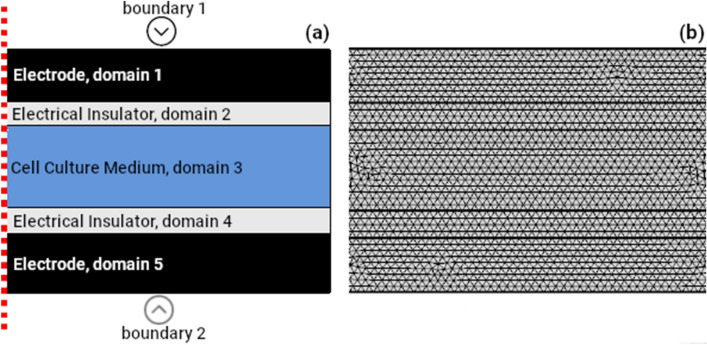
Axisymmetric representation of a typical CCoupled electric stimulation setup. (**a**) Domain identification of each layer, and electrical boundary conditions applied at the electrodes for FE analysis; (**b**) Example of a physics-controlled mesh obtained in COMSOL using the extra fine option.

#### Circuit simulator

As an alternative to the analytical approach, it is possible to use software packages for the simulation of analogue circuits to view the temporal variation of the current or of the voltage drop in the culture medium and to estimate the E-Field strength in the region of interest. We used the freely distributed program LTspice (LTspice LVII, Analog Devices, USA) to draw a circuit like the one illustrated in Fig. 1c considering only the 3 central sections since the impedance of the electrodes is negligible. The voltage waveform was specified as a sinusoidal waveform using the SINE option, as a trapezoidal pulse using the PULSE option, or as an arbitrary waveform using the PWL (piece-wise linear) option. After running the simulation, a LTspice probe tool was used to obtain the current through the resistive branch of the culture medium, from which the voltage drop and hence the E-Field were calculated. Note that for these simulations the resistive and capacitive impedances of the various layers were calculated based on a single, matching frequency obtained as outlined in Eq. (14). The two approaches should therefore provide the same estimates for the E-Field. Additionally, this implies that the simulator does not consider the full frequency spectrum of the waveform and so the predicted temporal variations are not exact but rather good approximations of the true variations.

#### Finite element analysis

If the geometry of the setup makes it difficult to estimate the resistance and capacitance of the various layers, then a numerical method that considers the specific features of the geometry should be applied to obtain accurate estimates of the E-Field. In this study, we used the Finite Element (FE) method for this purpose. Specifically, the commercial program COMSOL Multiphysics (version 5.2a, www.comsol.com, Stockholm, Sweden) was used to import the setup geometry defined in SolidWorks (version 2018, Dassault Systemes SolidWorks Corporation, France) and to create an extra fine, physics-controlled volume mesh. The Electric Currents interface of the AC/DC module was used to solve the underlying partial differential equations, with the direct solver MUMPS. This interface solves Laplace’s equation $\nabla \cdot (\sigma \nabla \phi ) = 0$, where $\phi$ is the electrostatic potential and $\sigma$ is the electric conductivity, and calculates the gradient of the scalar potential to determine the induced E-field. A Frequency Domain study was selected for sinusoidal voltages and a Time Dependent study for arbitrary waveforms. Note that no assumptions about the frequency spectrum of the voltage waveform are needed since the original waveform is used. The boundary conditions applied were Electric Potential and Ground for the two electrodes, Electric Insulation for other external boundaries and Current Conservation for internal boundaries. COMSOL can also handle ideal cylindrical geometries easily and efficiently as 2D axisymmetric models, as shown in Fig. 3.

**Figure 4:**
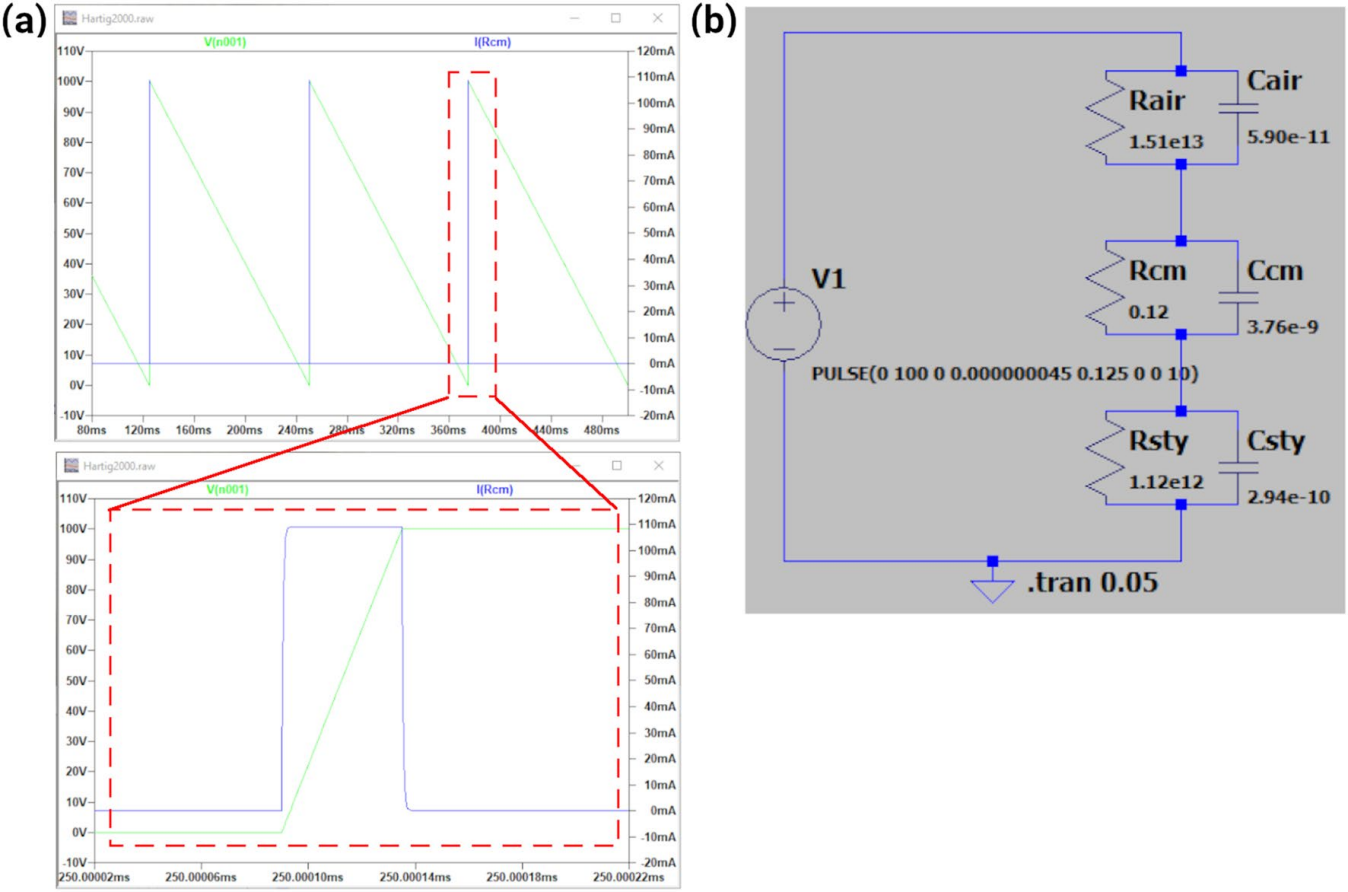
Example of LTspice analog circuit simulation for Hartig’s et al. setup^17^. Printscreens from the software environment: (**a**) digital probe tool visualizer showing the input potential signal (green line) and the electric current peak generated in the culture medium resistor (blue line). The bottom panel shows a detailed view of the rising edge for better visualization of the induced current; (**b**) Equivalent circuit model of Hartig’s setup drawn in LTspice. Abbreviations: R-resistor, C-capacitor, cm-culture medium, sty-polystyrene, V1-voltage source.

The three proposed approaches are based on well-known physics and well-established numerical methods and can produce accurate estimates of the E-Field for increasingly complex waveforms and geometries. They all assume that the quasi-electrostatic approximation holds.

### Selection of studies and theoretical validation

A bibliographic search was performed on ScienceDirect, Pubmed and Scopus databases to identify experimental studies using CCoupled stimulation. In order to narrow the search, only bone cell lineages were considered for this analysis taking into consideration our research group’s interest in bone tissue engineering. The following search sentence and keywords were considered:“(capacitive stimulation) AND (bone OR osteogenic OR osteogenesis) AND (in vitro)”, originating a total of 922 records, 881 in ScienceDirect, 30 in Pubmed and 11 in Scopus. After removal of duplicates, the remaining 883 records were screened considering the following exclusion (e) and inclusion (i) criteria:

1. Publications consisting in reviews or studies in vivo, or involving implants or prosthetic device;
2. Studies targeting biological tissues other than bone;
3. Studies targeting cellular processes other than proliferation and differentiation;
4. Studies using stimulation phenomena other than capacitive coupling;
5. The geometry of the experimental setup and voltage waveform must be reported in sufficient detail to allow the construction of a reasonably accurate model;
6. The cell culture chamber must be empty of any kind of construct and contain only cellular content and culture medium. This is because the presence of a scaffold can produce a highly non-uniform E-Field^22^;
7. The E-Field in the culture medium, measured or estimated, must be reported to allow a comparison with our model’s predictions.

A total of 16 records fulfilled all criteria. 4 additional records fulfilling all criteria were found by hand searching the reference lists in the 16 records mentioned previously. Eight different setups for capacitive stimulation are reported in these 20 records. They are listed below and were named after the first author of the oldest reference.

- Rodan et al., 1978, original description of this setup^7^;
- Korenstein et al., 1984, original description of this setup^12^, also used in^23–26^;
- Fitzsimmons et al., 1986, original description of this setup^6^, also used in^27,28^;
- Brighton et al. 1992, original description of this setup^15^, also used in^29–32^;
- Hartig et al., 2000, original description of this setup^17^, also used in^8^;
- Griffin et al., 2011, original description of this setup^18^, also used in^33^;
- Stephan et al., 2020, original description of this setup^19^;
- Khaw et al., 2021, original description of this setup^21^;

In all these studies the E-Field values reported were calculated, not measured.

**Figure 5:**
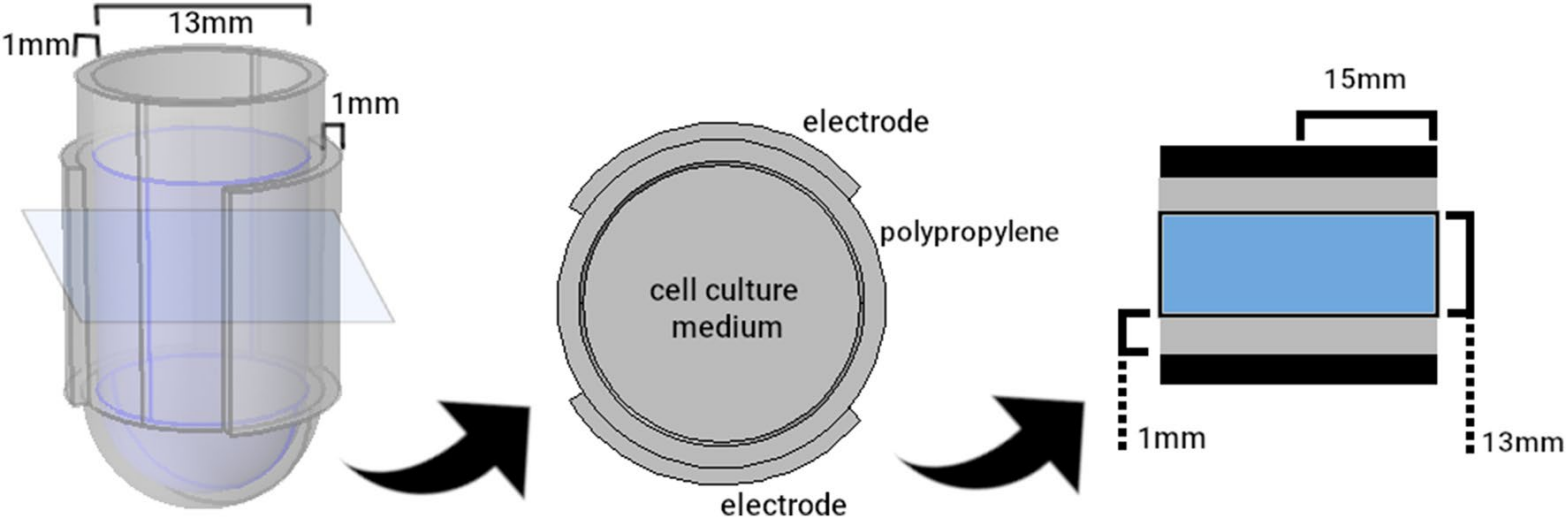
3D FE model created to replicate Rodan’s et al. setup^7^ and the geometrical approximation considered for its equivalent electronic circuit model.

For each one of these setups, the E-Field in the culture medium was calculated using the three approaches described in the previous section. The analytical solutions were implemented in Matlab and Python. The use of LTspice is exemplified in Fig. 4 with the asymmetric sawtooth voltage waveform applied in Hartig’s setup^17^. Rodan’s^7^, Stephan’s^19^ and Khaw’s^21^ setups are clearly different from the ideal layered cylindrical geometry. For these setups a realistic geometry was implemented in COMSOL. Fig. 5 shows the realistic model for Rodan’s setup [7], together with the layered cylindrical model with similar dimensions for analytical and circuit simulator calculations.

## Results

Modelling data like the dimensions of the setup, the values of the electrical properties of the materials, and details of the voltage waveform for all eight studies are compiled in Table 1. Note that the inverse of resistivity, i.e., the conductivity, $\sigma$, is listed in this table. Reasonable estimates for missing values were obtained from the literature cited in the table. The resistance, capacitance, and reactance of the three central layers of each setup were calculated based on these values and are listed in Table 2. Khaw’s setup is not listed in this table because at zero frequency the capacitive reactance of the insulators is infinite (Eq. 6), so the current through the setup and hence the E-Field in the culture medium will be zero. Note that values in this table are given with two decimal places for clarity but more decimal places were used in the calculations.

**Table 1:** Dimensions, electrical properties and waveforms for the setups modelled. $\epsilon _{r}$ is the relative permittivity, $\sigma$ is the conductivity.

Dimensions	Materials and electrical properties	Waveform
**Rodan** **et al.**,** 1978**^7^		
3D Model (Fig. 5), radius: 15 mm	*Layers 1,4:*	Positive trapezoidal pulse,
Radius: 15 mm	Copper: $\epsilon _{r}$=1; $\sigma$=5.998e7 S/m	1750 V amplitude,
*Layers 1,4:* Curved Electrodes	*layer 2:*	0.1 s pulse width,
Thickness - 1 mm	Polypropylene: $\epsilon _{r}$=2.1; $\sigma$=1e-16 S/m^10^	1.85 ms fall/rise time
*Layer 2:* Flask	*layer 3:*	
Thickness - 1 mm	Culture Medium: $\epsilon _{r}$=80.1; $\sigma$=1.5 S/m^11^	
*Layer 3:* Culture Medium		
Thickness - 13 mm		
**Korenstein et al.**, 1984^12^		
Radius: 27 mm		
*Layers 1,5:*	*layers 1,5:*	Negative trapezoidal pulse, 300 V,
Thickness - 1 mm	Copper: $\epsilon _{r}$=1; $\sigma$= 5.99 $\times$ ${10^{7}}$ S ${\hbox {m}^{-1}}$	500 V and 1300 V amplitude,
*Layer 2:*	*layer 2:*	25 $\mu \hbox {s}$ pulse width,
Thickness - 1.25 mm	Air: $\epsilon _{r}$=1.005; $\sigma$= 1.0 $\times$ ${10^{-14}}$ S ${\hbox {m}^{-1}}$	7 ns fall/rise time
*Layer 3:*	*layer 3:*	
Thickness - 2.25 mm	Culture Medium: $\epsilon _{r}$=74; $\sigma$= 1.5 S ${\hbox {m}^{-1}}$^11,12^	
*Layer 4:*	*layer 4:*	
Thickness - 1 mm	Polystyrene: $\epsilon _{r}$=2.5; $\sigma$= 6.7 $\times$ ${10^{-14}}$ S ${\hbox {m}^{-1}}$^13^	
**Fitzsimmons** **et al.**,** 1985**^6^		
Tadius: 26 mm		
*Layers 1,5:*	*layers 1,5:*	Sinusoidal wave,
Thickness - 1 mm	Metal Plates: $\epsilon _{r}$=1; $\sigma$= 5.99 $\times$ ${10^{7}}$ S ${\hbox {m}^{-1}}$	10 V amplitude, 10 Hz
*Layer 2:*	*layer 2:*	
Thickness - 10 mm	Air: $\epsilon _{r}$=1.005; $\sigma$= 1.0 $\times$ ${10^{-14}}$ S ${\hbox {m}^{-1}}$^14^	
*Layer 3:*	*layers 3:*	
Thickness - 7 mm	Culture Medium: $\epsilon _{r}$=80.1; $\sigma$=1.5 S/m^11^	
*Layer 4:*	*layer 4:*	
Thickness - 3 mm	Polystyrene: $\epsilon _{r}$=2.5; $\sigma$= 6.7 $\times$ ${10^{-14}}$ S ${\hbox {m}^{-1}}$^13^	
**Brighton** **et al.**,** 1992**^15^		
Radius: 16.5 mm		
*Layers 1,5:*	*layers 1,5:*	Sinusoidal Wave,
Thickness - 1 mm	Stainless Steel: $\epsilon _{r}$=1; $\sigma$=4.032e6 S/m	44.81 V amplitude,
*Layers 2, 4:*	*layers 2, 4:*	60 kHz
Thickness - 0.16 mm	No.1 Glass Coverslip: $\epsilon _{r}$=6.85; $\sigma$=1e-13 S/m^16^	
*Layer 3:*	*layers 3:*	
Thickness - 9.8 mm	Culture Medium: $\epsilon _{r}$=80.1; $\sigma$=1.5 S/m^11^	
**Hartig** **et al.**,** 2000**^17^		
Radius: 65 mm		
*Layers 1,5:*	*layers 1,5:*	Asymmetric sawtooth,
Thickness - 2 mm	High Grade Stainless Steel: $\epsilon _{r}$=1; $\sigma$= 0.14 $\times$ ${10^{7}}$ S ${\hbox {m}^{-1}}$	100 V peak-to-peak,
*Layer 2:*	*layer 2:*	45 ns risetime,
Thickness - 2 mm	Air: $\epsilon _{r}$=1.005; $\sigma$= 1.0 $\times$ ${10^{-14}}$ S ${\hbox {m}^{-1}}$^14^	62.5 ms falltime
*Layer 3:*	*layer 3:*	
Thickness - 2.5 mm	Culture Medium: $\epsilon _{r}$=80.1; $\sigma$=1.5 S/m^11^	
*Layer 4:*	*layer 4:*	
Thickness - 1 mm	Polystyrene: $\epsilon _{r}$=2.5; $\sigma$= 6.7 $\times$ ${10^{-14}}$ S ${\hbox {m}^{-1}}$^13^	
**Griffin** **et al.**, **2011**^18^		
Radius: 40 mm		
*Layers 1,5:*	*layers 1,5:*	Degenerate Wave,
Thickness - 1 mm	High Grade Steel: $\epsilon _{r}$=1; $\sigma$=5.99e7 S/m	160 mV peak-to peak,
*Layer 2:*	*layer 2:*	62.5 ms duration,
Thickness - 2 mm	Air: $\epsilon _{r}$=1.005; $\sigma$=1e-14 S/m^14^	16 Hz
*Layer 3:*	*layer 3:*	
Thickness - 4.7 mm	Culture Medium: $\epsilon _{r}$=80.1; $\sigma$=1.5 S/m^11^	
*Layer 4:*	*layer 4:*	
Thickness - 1 mm	Polystyrene: $\epsilon _{r}$=2.5; $\sigma$=6.7e-14 S/m^13^	
**Stephan** **et al.**,** 2020**^19^		
3D Model, radius: 16 mm		
*Layers 1,5:*	*layers 1,5:*	Sinusoidal Wave,
Thickness - 0.5 mm	Ti6Al4V: $\epsilon _{r}$=1; $\sigma$= 5.85 $\times$ ${10^{5}}$ S ${\hbox {m}^{-1}}$^20^	1.41 V and 0.141 V amplitude,
*Layer 2,4:*	*layer 2:*	60 kHz
Thickness - 1 mm	Polystyrene: $\epsilon _{r}$=2.5; $\sigma$= 6.7 $\times$ ${10^{-14}}$ S ${\hbox {m}^{-1}}$^13^	
*Layer 3:*	*layer 3:*	
Thickness - 32 mm	Culture Medium: $\epsilon _{r}$=80.1^11^; $\sigma$=1.6 S ${\hbox {m}^{-1}}$	
**Khaw** **et al.**, **2021**^21^		
3D Model, radius: 15 mm		
*layers 1,5:*	*layers 1,5:*	Constant Potential
Thickness - 1.75 mm	Electrode: $\epsilon _{r}$=1; $\sigma$=5.99e7 S/m	14.2 V and 28.4 V,
*Layer 2,4:*	*layer 2,4:*	
Thickness - 0.75 mm	Plastic: $\epsilon _{r}$=2; $\sigma$=6.7e-14 S/m^13^	
*Layer 3:*	*layer 3:*	
Thickness - 19.5 mm	Culture Medium: $\epsilon _{r}$=80; $\sigma$=1.7 S/m	
*Air sphere (3D model only):*	*air sphere (3D model only):*	
Radius: 120 mm	Air: $\epsilon _{r}$=1; $\sigma$=1e-14 S/m^14^

**Table 2:** Values of the resistances, capacitances, reactances and frequencies for the three-layer analogue circuit models of the CCoupled setups.

Layers	Resistance	Capacitance	Reactance	Frequency
**Rodan** **et al.**, **1978**^7^				
Layer 1 - Polypropylene	1.41 $${10^{16}} \Omega$$	1.31 $${10^{-11}}$$ F	− 1.41 $${10^{8}}$$ $$\Omega$$	86.03 $${\hbox {Hz}^{**}}$$
Layer 2 - Culture Medium	12.26 $\Omega$	3.86 ${10^{-11}}$ F	− 4.80 ${10^{7}}$ $\Omega$	
Layer 3 - Polypropylene	1.41 ${10^{16}} \Omega$	1.31 ${10^{-11}}$ F	− 1.41 ${10^{8}}$ $\Omega$	
**Korenstein et al., 1984**^12^				
Layer 1 - Air	5.46 $${10^{13}} \Omega$$	1.63 $${10^{-11}}$$ F	− 4.29 $${10^{2}}$$ $$\Omega$$	22.7 $${\hbox {MHz}^{**}}$$
Layer 2 - Culture Medium	0.65 $\Omega$	6.67 ${10^{-10}}$ F	− 0.10 ${10^{2}}$ $\Omega$	
Layer 3 - Polystyrene	6.52 ${10^{12}} \Omega$	5.07 ${10^{-11}}$ F	− 1.38 ${10^{2}}$ $\Omega$	
**Fitzsimmons et al., 1985**^6^				
Layer 1 - Air	4.71 ${10^{14}} \Omega$	1.89 ${10^{-12}}$ F	− 8.42 ${10^{9}}$ $\Omega$	10 Hz
Layer 2 - Culture Medium	2.20 $\Omega$	2.15 ${10^{-10}}$ F	− 7.40 ${10^{7}}$ $\Omega$	
Layer 3 - Polystyrene	2.11 ${10^{13}} \Omega$	1.57 ${10^{-11}}$ F	− 1.02 ${10^{9}}$ $\Omega$	
**Brighton et al., 1992**^15^				
Layer 1 - No.1 Glass Coverslip	1.87 ${10^{12}} \Omega$	3.24 ${10^{-10}}$ F	− 8.18 ${10^{3}}$ $\Omega$	60 kHz
Layer 2 - Culture Medium	7.79 $\Omega$	6.07 ${10^{-11}}$ F	− 4.37 ${10^{4}}$ $\Omega$	
Layer 3 - No.1 Glass Coverslip	1.87 ${10^{12}} \Omega$	3.24 ${10^{-10}}$ F	− 8.18 ${10^{3}}$ $\Omega$	
**Hartig et al., 2000**^17^				
Layer 1 - Air	1.51 $${10^{13}} \Omega$$	5.91 $${10^{-11}}$$ F	− 7.62 $${10^{2}}$$ $$\Omega$$	3.5 $${\hbox {MHz}^{**}}$$
Layer 2 - Culture Medium	0.13 $\Omega$	3.76 ${10^{-09}}$ F	− 1.20 ${10^{1}}$ $\Omega$	
Layer 3 - Polystyrene	1.12 ${10^{12}} \Omega$	2.94 ${10^{-10}}$ F	− 1.53 ${10^{2}}$ $\Omega$	
**Griffin et al., 2011**^18^				
Layer 1 - Air	3.98 ${10^{13}} \Omega$	2.24 ${10^{-11}}$ F	− 3.23 ${10^{8}}$ $\Omega$	22 Hz
Layer 2 - Culture Medium	0.62 $\Omega$	7.58 ${10^{-10}}$ F	− 9.54 ${10^{6}}$ $\Omega$	
Layer 3 - Polystyrene	2.97 ${10^{12}} \Omega$	1.11 ${10^{-10}}$ F	− 6.50 ${10^{7}}$ $\Omega$	
**Stephan et al., 2020**^19^				
Layer 1 - Polystyrene	1.86 ${10^{13}} \Omega$	1.78 ${10^{-11}}$ F	− 1.49 ${10^{5}}$ $\Omega$	60 kHz
Layer 2 - Culture Medium	24.87 $\Omega$	1.78 ${10^{-11}}$ F	− 1.49 ${10^{5}}$ $\Omega$	
Layer 3 - Polystyrene	1.86 ${10^{13}} \Omega$	1.78 ${10^{-11}}$ F	− 1.49 ${10^{5}}$ $\Omega$	

**Matched frequency.

The comparisons between the values of the E-Field in the culture medium reported in the original papers and those obtained using the three approaches described in this paper methods section are listed in Tables 3 and 4. Table 3 shows that there is a good agreement between the original reported values and our theoretical estimates in the case of the studies by Brighton et al.^15^, Hartig et al.^17^ and Stephan et al.^19^. In Brighton’s study, the reported E-Field values obtained using analytical and FE approaches^34^ agree with our estimates, for the two frequencies and applied voltages considered. The results are consistent with the fact that the E-Field is proportional to the applied voltage and that it is (approximately) proportional to the frequency. Hartig et al. reported a potential difference of 100 $\mu$V across the cell monolayer. We assumed that the thickness of the monolayer was 25 $\mu$m^35^, which yielded an E-Field estimate of 4 V ${\hbox {m}^{-1}}$. We also had to assume a risetime of 45 ns for the asymmetric sawtooth voltage waveform, based on the specifications sheet of the function generator (Hameg HM1881-2, www.farnell.com/datasheets/318574.pdf). Given these and other uncertainties, the agreement between the reported value (4.0 V ${\hbox {m}^{-1}}$) and our predictions (5.5 V ${\hbox {m}^{-1}}$) is acceptable. Stephan et al. estimated the electric field strength in a 3D model of a single well using the FE approach. Since the authors did not report the thickness of the petri dish wall, which separates the electrodes from the cell culture medium, we assumed a typical wall thickness of 1 mm. Despite this uncertainty, a good agreement between our predictions and the reported E-Field values was found. In general, small differences can be attributed to some of the model parameters not being described exactly in the original studies. Also, note that our three numerical approaches yield the same results, as expected.

**Table 3:** List of studies where an agreement was observed between the reported and predicted magnitude of the E-Field in the culture medium.

**Brighton et al., 1992**^15^** original setup, also reused in**^29–32^		
Waveform: sinusoidal	60 kHz, 44.81 V amplitude	10 Hz, 1.33 V amplitude
Reported Value	2.0 V ${\hbox {m}^{-1}}$	1.0 $\times {10^{-5}}$ V ${\hbox {m}^{-1}}$
Equivalent Electronic Circuit: Analytical	2.1 V ${\hbox {m}^{-1}}$	1.0 $\times {10^{-5}}$ V ${\hbox {m}^{-1}}$
Equivalent Electronic Circuit: LTspice (real waveform)	2.1 V ${\hbox {m}^{-1}}$	1.0 $\times {10^{-5}}$ V ${\hbox {m}^{-1}}$
Finite Element Analysis (FEA)	2.1 V ${\hbox {m}^{-1}}$	1.0 $\times {10^{-5}}$ V ${\hbox {m}^{-1}}$
**Hartig et al., 2000**^17^** original setup, also reused in**^8^		
Waveform: asymmetric sawtooth	45 ns rise-time, matched frequency 3.5 MHz, 100 V pk-pk	
Reported Value	4 V ${\hbox {m}^{-1}}$	
Equivalent Electronic Circuit: Analytical	5.5 V ${\hbox {m}^{-1}}$	
Equivalent Electronic Circuit: LTspice (real waveform)	5.5 V ${\hbox {m}^{-1}}$	
Finite Element Analysis (FEA)	5.5 V ${\hbox {m}^{-1}}$	
**Stephan et al., 2020**^19^** original setup**		
Waveform: sinusoidal	**60 kHz, 0.141 V amplitude**	**60 kHz, 1.41 V amplitude**
Reported Value	(2.5-3.5)x${10^{-4}}$ V ${\hbox {m}^{-1}}$	(2.5-3.5)x ${10^{-3}}$ V ${\hbox {m}^{-1}}$
Equivalent Electronic Circuit: Analytical	3.7 $\times {10^{-4}}$ V ${\hbox {m}^{-1}}$	3.7 $\times {10^{-3}}$ V ${\hbox {m}^{-1}}$
Equivalent Electronic Circuit: LTspice (real waveform)	3.7 $\times {10^{-4}}$ V ${\hbox {m}^{-1}}$	3.7 $\times {10^{-3}}$ V ${\hbox {m}^{-1}}$
Finite Element Analysis (FEA)	(2.2-6.0)x${10^{-4}}$ V ${\hbox {m}^{-1}}$	(2.5-3.5)x ${10^{-3}}$ V ${\hbox {m}^{-1}}$

The results concerning the studies where a discrepancy between the reported values and our estimates was observed are given in Table 4. In all cases, the E-Field in the culture medium was overestimated. In Rodan et al.^7^, no information on how the E-Field was estimated is provided. However, the slow risetime of 1.85 ms, which corresponds to a matched frequency of 86 Hz, seemed too low to produce an E-Field of 1.2 $\times {10^{5}}$ V ${\hbox {m}^{-1}}$. As the geometry of the setup differs from the layered cylindrical setup, a realistic model was implemented in COMSOL, as shown in Fig. 5. A layered cylindrical setup with similar dimensions was also designed to calculate resistances and capacitances for use in the analytical and circuit simulator approaches. All numerical approaches converged to a predicted field of about 6.0 $\times {10^{-3}}$ V ${\hbox {m}^{-1}}$, more than 7 orders of magnitude less than reported. Interestingly, the values obtained with the analytical and circuit simulator approaches are very similar to the value predicted by the FE analysis, even though they are based on different geometries.

**Table 4:** List of studies where a disagreement was observed between the reported and predicted magnitude of the E-Field in the culture medium.

**Rodan et al., 1978**^7^** original setup**			
Waveform: trapezoidal	1.85 ms rise-time, matched frequency 86 Hz, 1750 V amplitude		
Reported Value	1.2 $\times {10^{5}}$ V ${\hbox {m}^{-1}}$		
Equivalent Electronic Circuit: Analytical	5.9 $\times {10^{-3}}$ V ${\hbox {m}^{-1}}$		
Equivalent Electronic Circuit: LTspice (real waveform)	5.9 $\times {10^{-3}}$V ${\hbox {m}^{-1}}$		
Finite Element Analysis (FEA)	6.0 $\times {10^{-3}}$ V ${\hbox {m}^{-1}}$		
**Korenstein et al., 1984**^12^** original setup, also reused in**^23–26^			
Waveform: trapezoidal	7 ns rise-time, matched frequency 22.7 MHz, 300 V amplitude	7 ns rise-time, matched frequency 22.7 MHz, 500 V amplitude	7 ns rise-time, matched frequency 22.7 MHz, 1300 V amplitude
Reported Value	1.3 $\times {10^{3}}$ V ${\hbox {m}^{-1}}$	2.2 $\times {10^{3}}$ V ${\hbox {m}^{-1}}$	5.4 $\times {10^{3}}$ V ${\hbox {m}^{-1}}$
Equivalent Electronic Circuit: Analytical	1.5 $\times {10^{2}}$ V ${\hbox {m}^{-1}}$	2.6 $\times {10^{2}}$ V ${\hbox {m}^{-1}}$	6.7 $\times {10^{2}}$ V ${\hbox {m}^{-1}}$
Equivalent Electronic Circuit: LTspice (real waveform)	1.5 $\times {10^{2}}$ V ${\hbox {m}^{-1}}$	2.6 $\times {10^{2}}$ V ${\hbox {m}^{-1}}$	6.7 $\times {10^{2}}$ V ${\hbox {m}^{-1}}$
Finite Element Analysis (FEA)	1.5 $\times {10^{2}}$ V ${\hbox {m}^{-1}}$	2.6 $\times {10^{2}}$ V ${\hbox {m}^{-1}}$	6.7$\times {10^{2}}$ V ${\hbox {m}^{-1}}$
**Fitzsimmons et al., 1985**^6^** original setup, also reused in**^27,28^			
Waveform: sinusoidal	10 Hz, 10 V amplitude		
Reported Value	1.0$\times {10^{-5}}$ V ${\hbox {m}^{-1}}$		
Equivalent Electronic Circuit: Analytical	3.3$\times {10^{-7}}$ V ${\hbox {m}^{-1}}$		
Equivalent Electronic Circuit: LTspice (real waveform)	3.3$\times {10^{-7}}$ V ${\hbox {m}^{-1}}$		
Finite Element Analysis (FEA)	3.3$\times {10^{-7}}$ V ${\hbox {m}^{-1}}$		
**Griffin et al., 2011**^18^** original setup, also reused in**^33^			
Waveform: degenerate wave	Damped oscillation, matched frequency 22 Hz, 100 mV amplitude		
Reported Value	10 V ${\hbox {m}^{-1}}$		
Equivalent Electronic Circuit: Analytical	3.4$\times {10^{-8}}$ V ${\hbox {m}^{-1}}$		
Equivalent Electronic Circuit: LTspice (real waveform)	3.5 $\times {10^{-8}}$ V ${\hbox {m}^{-1}}$		
Finite Element Analysis (FEA)	3.6 $\times {10^{-8}}$ V ${\hbox {m}^{-1}}$		
**Khaw et al., 2021**^21^** original setup**			
Waveform: Steady Potential	Constant DC Potential, 14.2 V amplitude		Constant DC Potential, 28.4 V amplitude
Reported Value	100 V ${\hbox {m}^{-1}}$		200 V ${\hbox {m}^{-1}}$
Equivalent Electronic Circuit: Analytical	0 V ${\hbox {m}^{-1}}$		0 V ${\hbox {m}^{-1}}$

In Korenstein et al.^12^, only the relative permittivity of the various layers was taken into account, the electric resistivity of the culture medium was not considered. However, the resistance of the culture medium is the dominant factor affecting the E-Field in this layer. The other fundamental parameter that was missing was the risetime of the trapezoidal wave. We assumed a risetime of 7 ns based on the specifications sheet of the signal generator used Velonex 380 (https://www.testequipmentconnection.com/4603/Velonex380.php). This assumption and others regarding the conductivity of the various layers (Table 1) lead to an estimate for the E-Field in the culture medium of 154 V ${\hbox {m}^{-1}}$ for an applied voltage of 300 V by all numerical approaches used. This is one order of magnitude lower than the values reported by Korenstein et al. Estimates of the E-Field for voltages other than 300 V can be derived from the estimate presented here because the field is simply proportional to the applied voltage. In their paper, Korenstein et al. state that several experimental factors related to the electrical circuit distorted the voltage waveform, which suggest that the effective risetime may have been significantly longer than 7 ns. This would lead to a lower predicted E-field value.

The value of the E-Field reported in Fitzsimmons et al. (1.0$\times {10^{-5}}$ V ${\hbox {m}^{-1}}$)^6^ also differs significantly from our estimate (3.0 $\times {10^{-7}}$ V ${\hbox {m}^{-1}}$). This discrepancy arises from an incorrect estimate of the resistance of the culture medium: it seems that the resistivity of the culture medium (100 $\Omega$ cm) was incorrectly assumed to be equal its resistance (100 $\Omega$). We estimated the resistance of the culture medium to be about 30 times lower, which lead to a reduction in the estimated E-Field by the same factor. Also, Fitzsimmons et al. did not calculate the total impedance of the setup by summing the complex impedances of the various layers, which also lead to a small error in this value.

Griffin et al.^18^ used the same setup as Hartig et al. but they did not consider the time-varying nature of the applied voltage, the Degenerate Wave (DW), nor the conductive nature of the culture medium (see^33^, supplementary Figure$\_$S1.docx, File$\_$S2.docx). To get an accurate estimate of the field we obtained values for the conductivity of the various materials from the literature and digitized the DW from figure 1 in^33^. For use in our analytical approach, we estimated that a sine wave with a frequency of 22 Hz and 100 mV amplitude would have approximately the same maximum rate of change with time as the DW. The amplitude was taken to be 100 mV because this is the amplitude of the first (positive) deflection of the DW. Our numerical approaches estimated the E-Field in the culture medium to be approximately 3.5 $\times {10^{-8}}$ V ${\hbox {m}^{-1}}$, more than 8 orders of magnitude smaller than the value reported by Griffin et al.

Khaw et al.^21^ reported to have applied E-Fields of 100 V ${\hbox {m}^{-1}}$ and 200 V ${\hbox {m}^{-1}}$ in their capacitively-coupled bioreactor by applying constant potential differences of 14.2 V and 28.4 V respectively. These estimates were obtained with a FE 3D model, using the electrostatics interface to compute the E-Field. The selected set of equations does not take into account the conservation of currents and Ohm’s law, so it is insufficient to correctly model this CCoupled setup. As we stated in the methods section, the response of CCoupled systems is almost that of a capacitor, so the E-Field generated in the culture medium by continuous DC stimulation is effectively zero.

## Discussion

The results from our comparative analysis showed an overestimation of the E-Field in the majority of the works selected for this study. It also identified some wrong assumptions regarding the physics underlying the calculation of the E-Field in the culture medium. Based on these findings we provide some general advice for future aplications of capacitive coupled E-Field stimulation in TE.

In the three studies where there was a good agreement between the reported values and our theoretical estimates, we found that the electrical response of the setup was modelled correctly. Brighton et al.^15^ do not provide detailed information regarding the methods used to estimate the E-Field in the culture medium. However, in^34^ they stated in a footnote that “The E-Field and the current density were calculated on a macroscopic continuum basis by solving the boundary value problem constructed from Maxwell’s electromagnetic field equations. Comparable solutions were obtained using a two-dimensional closed-form solution technique and a three-dimensional computer-generated solution using finite element analysis”. This approach is similar to ours, which, together with reasonable estimates of the missing physical parameters, predicted almost the same value for the E-Field as reported in the paper. Hartig et al.^17^ based their E-Field estimates on an electric circuit described in^8^ and that is essentially the same as the one shown in Fig. 1 of this paper. Unfortunately, the values of the resistances and capacitances were not specified, nor was the duration of the rising edge of the saw-tooth voltage waveform. In addition, a potential difference across a cellular monolayer was reported, not the E-field in the culture medium. Despite these unknowns, the agreement between the reported value and our calculations was good. In Stephan et al.^19^ the E-Field was computed using the electric currents interface of the COMSOL FE software, based on the quasi-electrostatic approximation of Maxwell’s equations. This FE approach is the same as ours and despite the uncertainty due to the absence of the exact petri dish wall thickness, a good agreement was found with a typical value for this missing parameter.

Fitzsimmons et al.^6^ used an appropriate simplified electrical circuit to model the electrical response of the setup, but the resistance of the culture medium was likely overestimated, leading to an overestimate of the E-Field.

In the studies by Rodan et al.^7^, Korenstein et al.^12^, Griffin et al.^18^ and Khaw et al.^21^ the approach followed to estimate the E-Field was inappropriate because it did not consider the conductive nature of the culture medium or the temporal variation of the applied voltage. In CCoupled setups electric charge on the electrodes creates an E-Field in the culture medium which leads to the accumulation of charge of the opposite sign at the interface between the (conductive) culture medium and the insulator. This secondary charge distribution creates an E-Field that points in the opposite direction to the E-Field created by the charge on the electrodes and is such that, for a constant applied voltage (DC), the total E-Field in the culture medium would be zero.

In Rodan et al.^7^, the reported value of 1.166 $\times {10^{5}}$ V ${\hbox {m}^{-1}}$ was obtained by dividing the applied voltage (1750 V) by the distance between electrodes (15 mm) but this is physically incorrect given that the materials between the electrodes have very different conductivities and permittivities. In Korenstein et al.^12^, the resistive impedance of the culture medium is considerably lower than its capacitive reactance (Table 2), despite the high frequencies involved. It is therefore its resistive impedance that will determine the potential drop and hence the E-Field in the culture medium, thereby invalidating the assumption on which the calculations were based. In Griffin et al.^18^ the low resistive impedance of the culture medium is also ignored, resulting in an overestimation of the E-Field by eight orders of magnitude. In Khaw et al.^21^ the choice of the electrostatic interface for the FE analysis, which cannot take into account currents in conducting media, is the reason why a non-zero E-field was wrongly predicted.

The analysis of these eight studies, highlights the importance of accurate and detailed reporting of the physical parameters of the setup (dimensions, electrical properties) and the voltage waveform (particularly, risetimes of sharp edges) to enable replication of the electrical stimulation. When this information is available, a number of numerical approaches can produce sufficiently accurate estimates of the E-Field in the culture medium. The resulting computational model of the setup constitutes its digital twin, which has several useful characteristics. It can be shared, and it can be used to investigate the effect of changes in the setup parameters and voltage waveform on the E-Field in the culture medium. It can also be used to determine the changes in the setup required to achieve the desired E-Field. CAD files are a practical way of documenting and sharing the geometry of the setup.

Regarding the choice of the numerical approach, an analytical solution based on the simple series C-R-C circuit shown in Fig.1d should provide a useful first estimate of the E-Field in most cases. This approach can be extended to non-sinusoidal waveforms by estimating the frequency associated with the fastest rising or falling edges of the applied voltage. Alternatively, a circuit simulator like LTspice can provide a graphical illustration of the temporal variation of the E-Field in the culture medium for arbitrary waveforms. Note, however, that a single frequency must still be chosen to calculate the capacitance of the insulating layers. Also, a very good approximation to the temporal variation of the E-Field can also be obtained by simply plotting the first derivative of the voltage waveform with respect to time (Eq. 13).

If the geometry of the setup differs significantly from the ideal coaxial geometry of constant section that is assumed in the analytical solution, then the FE method may be used to take into account the complexity of the geometry. However, even in the case of the geometry implemented in Rodan et al.^7^ and shown in Fig. 5a, it was possible to estimate the E-field using a simple cylindrical (coaxial) model (Fig. 5b) and still obtain almost identical values for the E-Field (Table 4). Another advantage of the FE method is that it makes no assumptions about the frequency spectrum of the applied voltage, which is not the case for the analytical and circuit simulator approaches. Nonetheless, our predictions of the E-Field for the eight studies analysed (Tables 3 and 4) are practically the same for all three independent approaches (less than 3% deviation from the mean value of the three prediction methods).

The E-Field values reported in the selected studies ranged from 1.0$\times {10^{-5}}$ V ${\hbox {m}^{-1}}$ to 1.0$\times {10^{5}}$ V ${\hbox {m}^{-1}}$. According to our calculations the actual range of applied fields was 1.0$\times {10^{-8}}$ V ${\hbox {m}^{-1}}$ to 1.0$\times {10^{2}}$ V ${\hbox {m}^{-1}}$, still a range of 10 orders of magnitude. This is explained in part by the wide range of frequencies used, from Hz to MHz, and the fact that E-Field strength is proportional to frequency in the setups described.

The effect of electrical stimulation on cell response is likely to be frequency dependent. For example, Brighton et al.^15^ failed to reproduce the effects on cell proliferation reported in Fitzsimmons et al.^6^ at 10 Hz and an E-Field strength of 1.0$\times {10^{-5}}$ V ${\hbox {m}^{-1}}$ (and note that Fitzsimmons probably applied a field some 30 times weaker). On the other hand, Krueger et al.^36^ have recently reported an effect on chondrocytic differentiation capacity with fields of 5.2$\times {10^{-6}}$ V ${\hbox {m}^{-1}}$ and 5.2$\times {10^{-5}}$ V ${\hbox {m}^{-1}}$ at a frequency of 1 kHz. Thus, optimization of CCoupled electrical stimulation protocols should consider E-Field strength and frequency as independent parameters.

Overall, the results presented in Tables 3 and 4 show that the analytical and circuit simulator approaches outlined previously may give an estimate of the E-Field intensity with sufficient accuracy for most purposes. In this work we have assumed a homogeneous culture medium. The presence of a scaffold can introduce local variations of the E-Field (hotspots, coldspots) that may introduce localized effects on the cell culture^22^. In this case, the FE method should be applied to take into consideration the complex geometry of the scaffold.

In this study we analyzed the E-Field in eight CCoupled setups that were used in twenty studies, published between 1978 and 2021. The E-Field was correctly estimated in only 3 out of 8 setups and 8 out of 20 studies. We limited our analysis to bone and osteogenesis related studies but similar trends will probably be found in applications involving other tissues. Of course, the methods outlined here can used to predict the E-Field in CCoupled setups for electric stimulation of cell cultures of any type.

Based on the analytical approach presented in this work, we have developed an E-Field Calculator for CCoupled Systems with a layered cylindrical geometry. This calculator is free, open-source and is publicly available for download from the Zenodo platform (CCoupled E-Field Calculator, https://doi.org/10.5281/zenodo.5897226). More details about the E-Field calculator and its operation are available in the supplementary materials.

Even though the laws of physics enable a reasonably accurate prediction of the E-field in the culture medium, the model should still be validated experimentally. Ideally, the E-field strength in the culture medium should be measured, but it may be difficult to do it correctly and accurately in most setups. Alternatively, the applied voltage and the current through the setup should be measured and reported. The ratio of these two quantities gives the total impedance of the setup, which can be compared with the value predicted by the model.

## Conclusion

This work has shown a predominant overestimation of the E-Field applied in capacitively coupled ES studies. Furthermore, the reported E-Fields were calculated and not measured at any stage of the experimental protocol. Errors in E-Field calculation may have lead to wrong conclusions regarding the influence of the E-Field on cell homeostasis, growth and differentiation. In future CCoupled studies, the setup for electric stimulation should be designed based on a numerical approach, such as the ones outlined in this paper, to estimate the E-Field in the culture medium previous to the construction of the experimental setup. This digital twin should contain all the information necessary to reproduce the experimental setup and should be made available on publication of the study. In addition, the predictions from the model should be validated to the largest possible extent, to confirm the E-Field characteristics that originated the cellular effects observed. These improvements would be crucial steps towards understanding how CCoupled stimulation modulates cellular behaviour, allowing to further optimize stimulation protocols for an effective translation of this technique to the clinical context.

## Supplementary Information

Supplementary Information.

## Data Availability

The CCoupled E-Field calculator can be downloaded and installed in two ways: (1) Download all the project files from Zenodo (https://doi.org/10.5281/zenodo.5897226) into your destination folder, and with a Python IDE with all the required dependencies, run the script named “CCoupledCalculator.py”; (2) Inside the same Zenodo project directory, download the *.zip archive “CCoupledCalculator.zip” that contains an executable file for Windows OS. This file was generated with PyInstaller, and by running it, a standalone version of this E-Field calculator is launched without the need to install python or its dependencies.
