# Author Correction: How to correctly estimate the electric field in capacitively coupled systems for tissue engineering: a comparative study

**DOI:** 10.1038/s41598-022-16724-z

**Published:** 2022-07-22

**Authors:** João Meneses, Sofia Fernandes, Nuno Alves, Paula Pascoal-Faria, Pedro Cavaleiro Miranda

**Affiliations:** 1 Centre for Rapid and Sustainable Product Development from the Polytechnic of Leiria, Leiria, Portugal; 2 Mathematics Department of Faculty of Management and Technology from the Polytechnic of Leiria, Leiria, Portugal; 3 IBEB, Faculdade de Ciências, Universidade de Lisboa, 1749-016 Lisboa, Portugal

Correction to: *Scientific Reports* 10.1038/s41598-022-14834-2, published online 30 June 2022

In the original version of this Article Paula Pascoal‑Faria and Pedro Cavaleiro Miranda were omitted as jointly supervising authors.

The original Article has been corrected.

