# Supplementary material for: How to correctly estimate the electric field in capacitively coupled systems for tissue engineering: a comparative study
Source: Sci Rep. 2022 Jun 30;12:11049. doi: 10.1038/s41598-022-14834-2 (PMC9247067; doi:10.1038/s41598-022-14834-2)
Supplement: Supplementary file 1 — Supplementary Information. [file 41598_2022_14834_MOESM1_ESM.pdf]

How to correctly estimate the Electric Field in Capacitively Coupled Systems for Tissue Engineering: A Comparative Study

João Meneses*, Sofia Fernandes, Nuno Alves,
Paula Pascoal-Faria, and Pedro Cavaleiro Miranda
*Corresponding Author

E-Field Calculator for CCoupled Systems. This calculator predicts the electric field (E-Field) in the culture medium of a cylindrical capacitively-coupled setup, based on the analytical approach published in [insert full reference with DOI]. The open-source license and disclaimers may be consulted by clicking on the “About” button, shown in fig. A1. By using the Calculator the user agrees with the license and disclaimers.

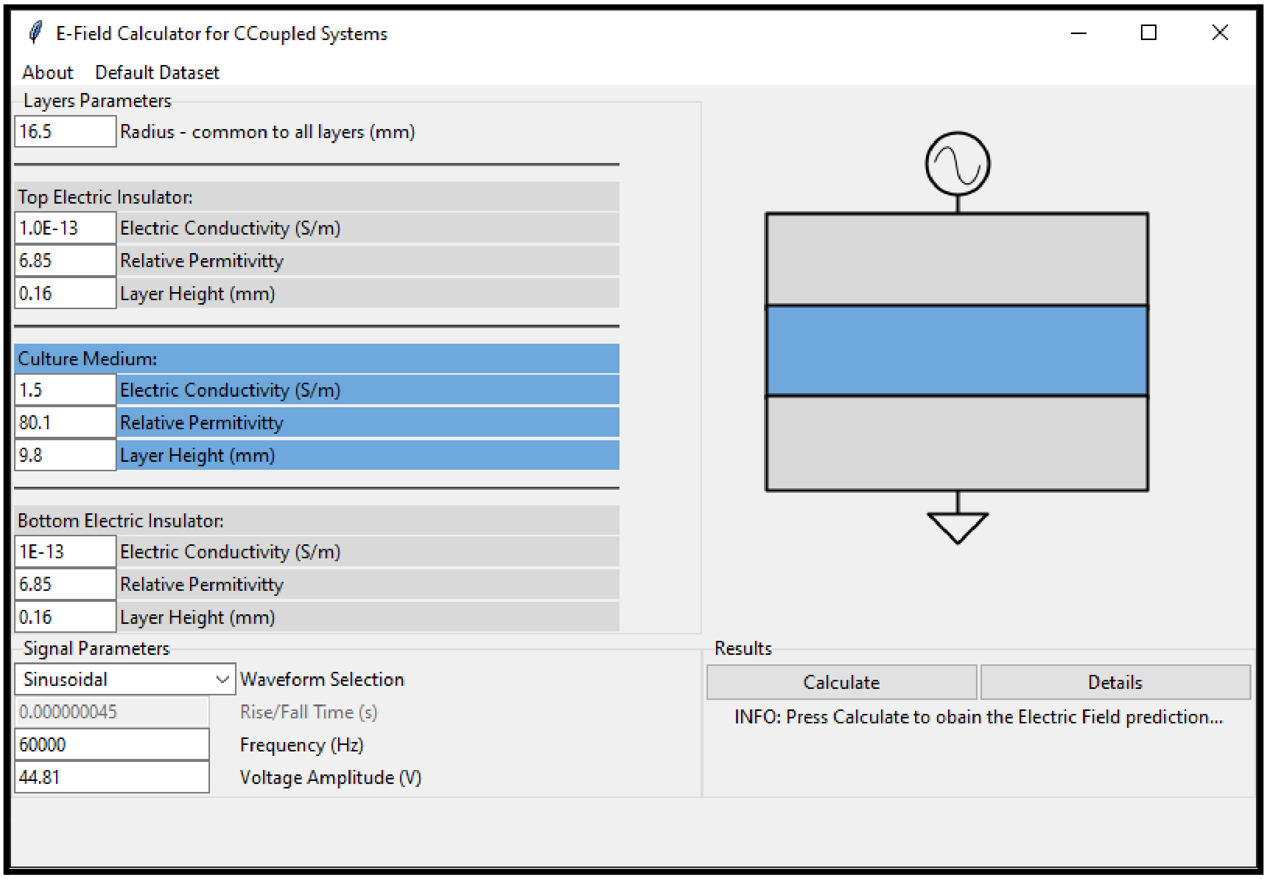

Figure A1: Graphical user interface of the E-field Calculator. The default values correspond to the setup described in Brighton et al., 1992

The graphical user interface of the E-field Calculator is shown in fig. A1. It comes prefilled with the values that correspond to the setup described in Brighton et al., 1992.

The fields in white are editable and should be changed to reflect as close as possible the user's setup. Values for the electric conductivity and relative permittivity of the materials can be obtained from the manufacturer or approximate generic values can be sought on the internet. The waveform can be sinusoidal, in which case its frequency and the amplitude (half of the peak-to-peak value) must be specified, or a linear ramp, in which case the rise-time and the height of the ramp (in "voltage amplitude") must be specified. Clicking on the "Calculate" button, in the bottom right-hand corner, will run the calculation and display the value of the E-field in the culture medium (Em) in that box, see fig. A2.

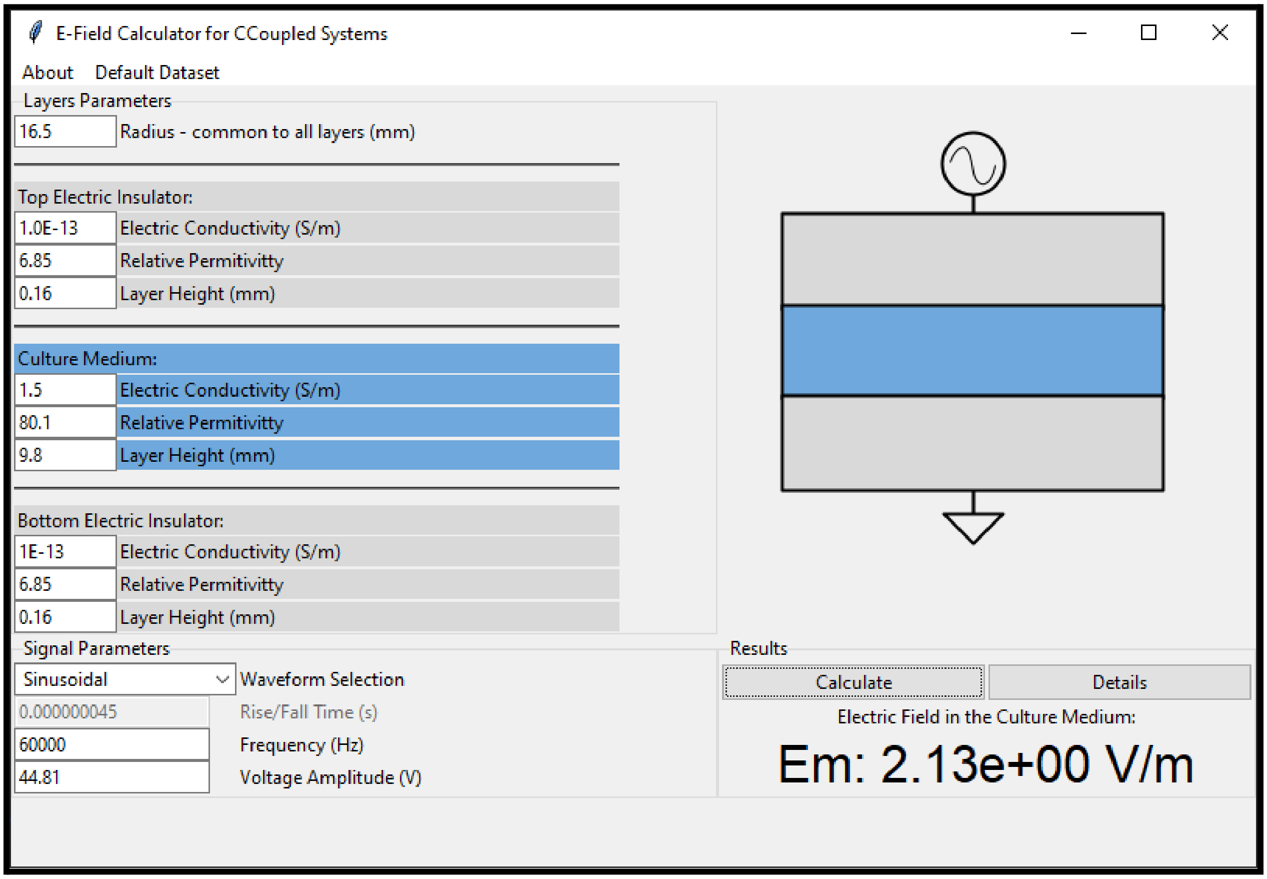

Figure A2: The graphical user interface displaying the magnitude of the E-Field in the culture medium in the bottom right-hand corner for the setup in Brighton et al., 1992.

Further details about intermediate results, including the electric current (I) in the system, can be obtained by clicking on the "Details" button after performing the calculation. The detailed information will be shown in a pop-up window, as shown in fig. A3.

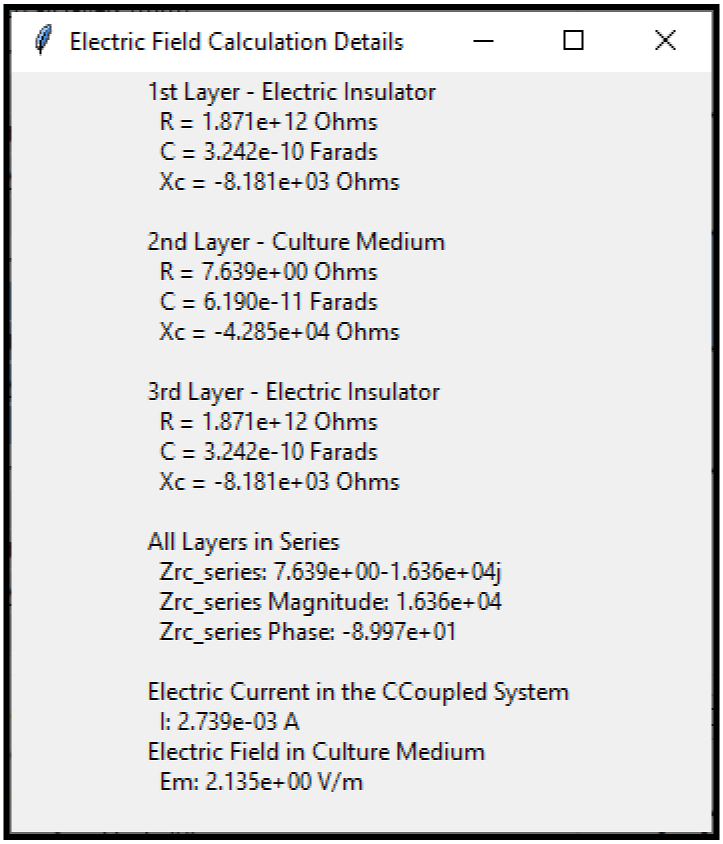

Figure A3: Pop-up window with details of the impedance calculations, the current in the setup and the electric field in the culture medium for the setup in Brighton et al., 1992 (R - resistance, C - capacitance, Xc - capacitive reactance, Z - impedance).

The user can also prefill the calculator with the values corresponding to the setup in Hartig et al., 2000 by picking the appropriate "Default Dataset" in the top menu, as shown in fig. A4. In this case, the waveform is an asymmetric sawtooth with a rise time estimated at 45 ns.

To run the E-Field calculator the user has two options: 1) Download all the project files from Zenodo (https://doi.org/10.5281/zenodo.5897226) into your destination folder, and with a Python IDE with all the required dependencies, run the script named “CCoupledCalculator.py”; 2) Inside the same Zenodo project directory, download the *.zip archive “CCoupledCalculator.zip” that contains an executable file for Windows OS. This file was generated with PyInstaller, and by running it, a standalone version of this E-Field calculator is launched without the need to install python or its dependencies.

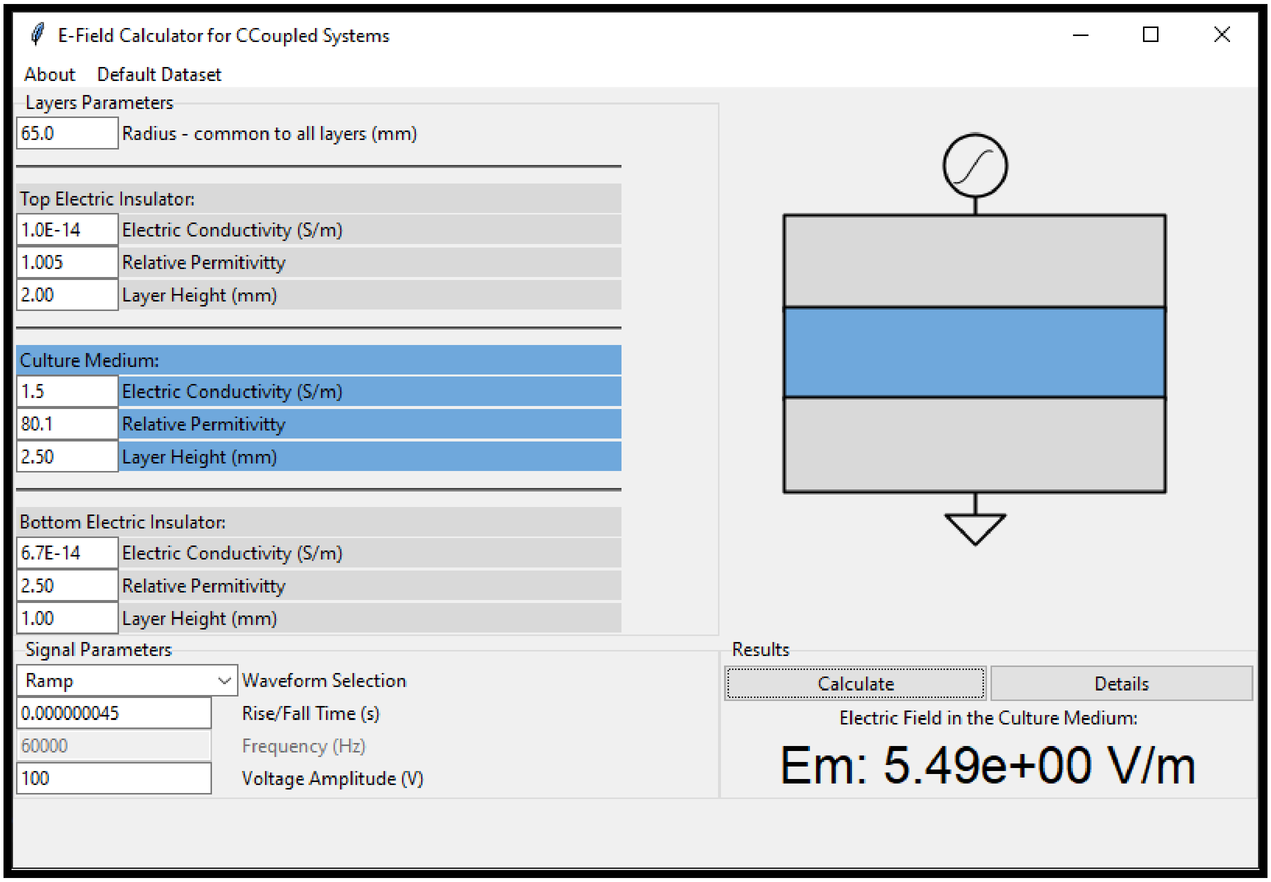

Figure A4: The graphical user interface displaying the magnitude of the E-Field in the culture medium in the bottom right-hand corner for the setup in Hartig et al., 2000.
